# Non‐invasive diagnosis of cirrhosis and long‐term disease monitoring by transient elastography in patients with Wilson disease

**DOI:** 10.1111/liv.14368

**Published:** 2020-01-22

**Authors:** Rafael Paternostro, Jan Pfeiffenberger, Peter Ferenci, Albert F. Stättermayer, Rudolf E. Stauber, Fritz Wrba, Thomas Longerich, Karoline Lackner, Michael Trauner, Arnulf Ferlitsch, Thomas Reiberger, Karl Heinz Weiss

**Affiliations:** ^1^ Division of Gastroenterology and Hepatology Department of Internal Medicine III Medical University of Vienna Vienna Austria; ^2^ Department of Internal Medicine IV University Hospital Heidelberg Heidelberg Germany; ^3^ Division of Gastroenterology and Hepatology Department of Internal Medicine Medical University of Graz Graz Austria; ^4^ Department of Pathology Medical University of Vienna Vienna Austria; ^5^ Institute of Pathology University Hospital Heidelberg Heidelberg Germany; ^6^ Institute of Pathology Medical University of Graz Graz Austria; ^7^ Ludwig Boltzmann Institute for Rare and Undiagnosed Diseases Vienna Austria; ^8^ CeMM Research Center for Molecular Medicine of the Austrian Academy of Sciences Vienna Austria

**Keywords:** cirrhosis, non‐invasive fibrosis scores, transient elastography, Wilson disease

## Abstract

**Background & Aims:**

The value of liver stiffness measurement (LSM) by transient elastography (TE) for non‐invasive fibrosis staging and disease monitoring has not been established in patients with Wilson disease (WD).

**Methods:**

Liver stiffness measurement by TE and non‐invasive fibrosis scores (APRI, FIB‐4) were analysed from 188 WD patients with liver biopsy (LBX). Longitudinal LSM was performed in 128 (68.1%) patients.

**Results:**

One hundred and eighty‐eight patients (mean age: 35 ± 14 years, 54.8% women; 27.1% with histological cirrhosis) were studied. Forty‐four[23.4%] patients were recently diagnosed with WD, while 144[76.6%] were previously diagnosed (>1 year between LBX and LSM). Overall, LSM (11.3 vs 6.1 kPa, *P* < .001), APRI (0.72 vs 0.38, *P* < .001) and FIB‐4 (1.54 vs 0.89, *P* < .001) were higher in cirrhotic than in non‐cirrhotic patients. This was even more pronounced in recently diagnosed patients (35.2 kPa vs 6.4 kPa, *P* < .001). Accuracy for diagnosing cirrhosis at an LSM cut‐off ≥9.9 kPa was better in recently diagnosed (PPV: 74%, NPV: 100%) vs previously diagnosed (PPV: 53%, NPV: 82%) patients. Recently diagnosed patients had higher Area Under the Curve (AUC) for APRI (0.79 vs 0.61) and FIB‐4 (0.84 vs 0.65) than previously diagnosed patients. At APRI <1.5 and FIB‐4 <3.25 cirrhosis was ruled out with a specificity of 93% and 95% respectively. During a median follow‐up of 46 (24‐66) months, only 5.9% (5/85) of non‐cirrhotic WD patients showed progression to cirrhotic LSM values, while 30.8% (4/13) of cirrhotic WD patients showed LSM suggestive of cirrhosis regression.

**Conclusion:**

TE‐based LSM ≥9.9 kPa accurately identifies cirrhosis in WD patients. Next to TE‐LSM <9.9 kPa, APRI <1.5 and FIB‐4 <3.25 values assist to non‐invasively rule out cirrhosis. LSM remains stable in most non‐cirrhotic patients on WD therapy, while one‐third of cirrhotic patients present clinically relevant decreases in LSM.

**Key points**

- Transient elastography (TE) is a validated non‐invasive tool for staging fibrosis and monitoring disease progression in patients with chronic liver disease, nevertheless, its role in rare aetiologies such as Wilson disease (WD) has not been established.
- We investigated the value and applicability of TE‐based liver stiffness measurement (LSM) in 188 patients with biopsy‐proven WD.
- Our data suggest that TE and non‐invasive fibrosis scores are useful to identify cirrhosis in patients with recently diagnosed WD.
- In most WD patients, LSM by TE remained stable over time, while a significant proportion (30.8%) of cirrhotic patients showed improvements of LSM under WD therapy. LSM only worsened in 5.6% of recently diagnosed patients and in 8.5% of previously diagnosed patients.

## 1 INTRODUCTION

Wilson disease (WD) is a genetic disease of impaired biliary copper excretion resulting in accumulation of copper in the liver and extrahepatic tissues.^1^, ^2^, ^3^ The key features of WD are all forms of liver disease, neuropsychiatric disturbances, Kayser‐Fleischer rings and acute episodes of haemolysis that often associate with acute liver failure. The clinical presentation is highly variable and patients may become symptomatic at any age,^4^ and about half of the patients develop cirrhosis.^5^

Currently there is an ongoing discussion on how to evaluate the efficacy of new treatments of WD. Similar to Non‐alcoholic steatohepatitis (NASH),^6^ there are no validated study endpoints in WD. Due to the various phenotypic presentation validated endpoints for neurologic and hepatic disease are needed. Until recently, liver biopsy was the only tool to stage fibrosis/cirrhosis in any patient with liver disease. Novel non‐invasive staging‐methods, such as transient elastography (TE) and blood‐based fibrosis scores such as APRI^7^ or FIB‐4^8^ have been developed. However, both TE and APRI/FIB‐4 have been mostly validated for fibrosis staging in viral liver disease^7^, ^9^, ^10^, ^11^, ^12^ and in non‐alcoholic fatty liver disease.^10^, ^13^, ^14^ Data on non‐invasive methods to stage fibrosis in WD are scarce.^15^ After long discussions with Food and Drug Administration (FDA) and European Medicines Agency (EMA) it became clear that our armamentarium to predict the outcome of liver disease (ie surrogate endpoints) in WD is very limited. One of the proposals was to assess the value of repeated liver stiffness measurements (LSM) by TE. To date, only two small studies^16^, ^17^ have investigated TE‐based LSM in adult WD patients, however, both included very few patients with cirrhosis.

Therefore the aim of our retrospective international multicentre study was to evaluate the value of TE‐based LSM, APRI and FIB‐4 as non‐invasive tools for fibrosis assessment in patients with liver disease:

Firstly, we assessed the diagnostic accuracy of TE‐based LSM, APRI and FIB‐4 for non‐invasive detection of cirrhosis in WD patients undergoing liver biopsy as reference.

Secondly, we evaluated the course of TE‐based LSM in WD patients in daily clinical practice and its potential use in prospective phase 3 trials.

## 2 METHODS

### 2.1 Patients

**Figure 1:**
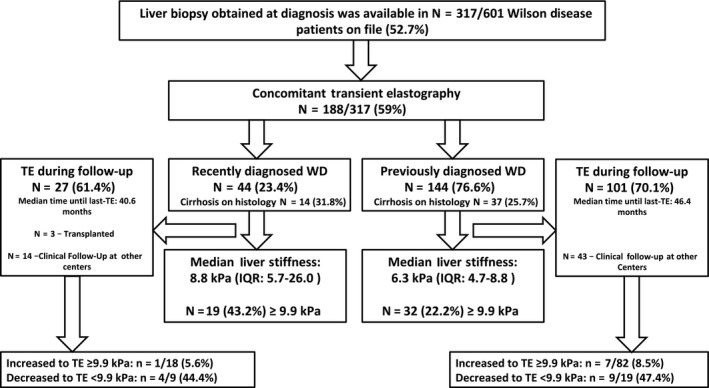
Flow chart

One hundred and eighty‐eight WD patients from three tertiary care hospitals (Medical University of Vienna, Medical University of Heidelberg, Medical University of Graz) with available data on liver histology and TE values were studied. Patients attended the WD clinic at least once a year. At each control, patients were examined physically and standard laboratory parameters including serum copper, serum ceruloplasmin by nephelometry, and 24 hours urinary copper excretion were recorded. As soon as the TE equipment became available, TE examinations were included. Monitoring of the patients was based on clinical findings, stable liver function tests and 24‐hour urinary copper excretion. See Figure 1 for a detailed study flow chart.

Since many patients attended the clinic for many years before TE became available the patients were divided in two groups: ‘ Recenty diagnosed’ WD patients had LSM and liver biopsy within <1 year after considering the diagnosis of WD (treatment was recently established in 14 [31.8%]; median duration of 7.5 months). ‘Previously diagnosed’ patients were under treatment for >1 year after the diagnostic biopsy before the first TE was done. Among the 144 ‘Previously diagnosed’ patients (76.6%) under treatment (d‐penicillamin 78 [54.2%], trientine 37 [25.7%], zinc 16 [11%], combination therapies 7 [4.9%], unknown 3 [2.1%]), three (2.1%) patients participated in the phase 2 trial using bis‐choline tetrathiomolybdate (TTM, WTX101^18^).

The following data were recorded: Phenotypic presentation (hepatic, neurologic), type and duration of treatment, standard laboratory data to calculate APRI^7^ and FIB‐4^8^ and endoscopic data (available in 49 of 51 cirrhotic patients, no gastroscopy was performed in non‐cirrhotic patients). Follow‐up LSM was recorded in all patients if available and the same quality criteria as for the baseline TE were used (see below). Decreasing LSM was defined as reduction by at least −10% and/or −1 kPa, stable LSM as values ±10% and increasing LSM as an increase of at least +10% and/or +1 kPa from BL‐ to last LSM. Furthermore the proportion of patients whose last LSM measurement overstepped respectively fell below the threshold of 9.9 kPa are reported (threshold was derived by the study data). Transaminase levels and platelet count to calculate APRI and FIB‐4 were recorded in recently diagnosed patients during follow‐up.

### 2.2 Transient elastography

Liver stiffness measurement was performed using TE (FibroScan, Echosense, France) in a supine position with the right arm resting behind the head of the patient and under fasting conditions for at least four hours. At least ten valid measurements were performed in each patient and the median value was then taken into account. Only values with an IQR/M <30% were used for statistical analysis.^19^

### 2.3 Liver histology

Liver biopsies (LBX) were performed at the time of diagnosis or histological findings were obtained from the explanted liver. In many patients the date of biopsy predated the first elastography. Biopsies were read by the local hepatopathologist (FW, TL, KL) as part of the clinical routine. For this study, we classified biopsy results from the written report as: normal, steatosis without significant fibrosis, fibrosis (F1‐F3) and cirrhosis.

### 2.4 Statistics

Continuous variables were reported as mean ± standard deviation or median (Interquartile Range[IQR]), and categorical variables were reported as number (n) of patients with the certain characteristic (proportion of patients with the certain characteristics [%]). Student t test was used for group comparisons of normally distributed data and Mann‐Whitney‐U test where data was not normally distributed. Pearson's Chi‐Square test or Fisher's exact test was performed to conduct group comparisons, as appropriate. The area under receiver operating characteristic curve was used to assess the diagnostic/predictive value of quantitative variables for a dichotomous outcome (nominal variable). Youden Index was used to determine optimal cut‐offs for cirrhosis for TE, APRI and FIB‐4. Positive as well as negative predictive values (PPV and NPV) were calculated. Binary logistic regression analysis was performed to find factors independently associated with cirrhotic LSM values ≥9.9 kPa. First a univariable model was conducted and then the preselected variables were put into two multivariable models (two models were chosen because of multicollinearity between APRI and FIB‐4 scores).

The IBM SPSS 24.0 statistic software (SPSS Inc, Armonk, NY) was used for all statistical analysis.

### 2.5 Ethics

This study was approved by the local ethics committees and performed in accordance to the current version of Helsinki Declaration. Since this was a retrospective study, no informed consent was needed.

## 3 RESULTS

One hundred and eighty‐eight patients were included in the final analysis. Laboratory data were missing in six patients, hence data on APRI and FIB‐4 were only available in 182 patients (97%) when LSM was performed. Histologic diagnosis of cirrhosis was made in 51 (27.1%) patients. Forty‐four (23.4%) patients were classified as ‘recently diagnosed’ and they were significantly younger than previously diagnosed patients (30 ± 13 vs 36 ± 14, *P* = .008). Upper‐gastrointestinal endoscopy was available in 49 of 51 patients with cirrhosis. Interestingly recently diagnosed cirrhotics had more often gastroesophageal varices than previously diagnosed cirrhotics (7 [63.6%] vs 4 [36.5%], *P* = .002). LSM (8.8 vs 6.3, *P* = .001), AST levels (58 vs 30, *P* < .001) and ALT levels (40 vs 27, *P* = .001) as well as APRI (0.82 vs 0.38, *P* < .001) were all higher in recently diagnosed patients. Further main patient characteristics are listed in Table 1 and Figure 1.

**Table 1:** Patient characteristics at the time of first LSM measurement

	All (n = 188)	Recently diagnosed (n = 44)	Previously diagnosed (n = 144)	*P*‐value
Mean age, mean ± SD (y)	35 ± 14	30 ± 13	36 ± 14	.008
Gender, n, (%)				
Male	85 (45.2%)	24 (28.2%)	61 (71.8%)	.155
Female	103 (54.8%)	20 (19.4%)	83 (80.6%)	
Centre, n, (%)				
Vienna	59 (31.4%)	15 (25.4%)	44 (74.6%)	.660
Heidelberg	120 (63.8%)	26 (21.7%)	94 (78.3%)	
Graz	9 (4.8%)	3 (33.3%)	6 (66.7%)	
Liver Biopsy, n, (%)				
No cirrhosis	137 (72.9%)			
Normal/minimal changes	41 (21.8%)	9 (22%)	32 (78.0%)	.840
Steatosis	37 (19.7%)	9 (24.3%)	28 (75.7%)	
Fibrosis F2‐F3	59 (31.4%)	12 (20.3%)	47 (79.7%)	
Cirrhosis	51 (27.1%)	14 (27.5%)	37 (72.5%)	
Neurologic symptoms at LBX, n, (%)	32/170 (18.8%)	9 (28.1%)	23 (71.9%)	.787
LSM by TE, median (IQR)	6.8 (4.9‐10.0)	8.8 (5.7‐26.0)^b^	6.3 (4.7‐8.8)^a^	.001
AST U/L, median (IQR)	34 (23‐61)	58 (29‐83)	30 (21‐43)	<.001
ALT U/L, median (IQR)	29 (21‐46)	40 (26‐109)	27 (20‐38)	.001
PLT G/L, mean ± SD	212 ± 88	194 + 92	218 ± 87	.142
APRI, median (IQR)^d^	0.42 (0.25‐0.87)	0.82 (0.33‐1.72)	0.38 (0.23‐0.76)	<.001
FIB‐4, median (IQR)^d^	1.06 (0.61‐2.04)	1.40 (0.57‐2.87)	1.04 (0.62‐1.65)	.164
Oesophageal varices, n(%)^c^				
No	38 (77.6%)	6 (15.8%)	32 (84.2%)	.002
Yes	11 (22.4%)	7 (63.6%)	4 (36.4%)	

Abbreviations: IQR, Interquartile‐Range; LSM, liver stiffness measurement; TE, transient elastography.

a First examination.

b At biopsy.

c Evaluated in n = 49 patients with a clinical suspicion of cirrhosis or LSM result suggestive of cirrhosis.

d Available in 182 patients.

### 3.1 Diagnostic accuracy of TE to diagnose cirrhosis

**Figure 2:**
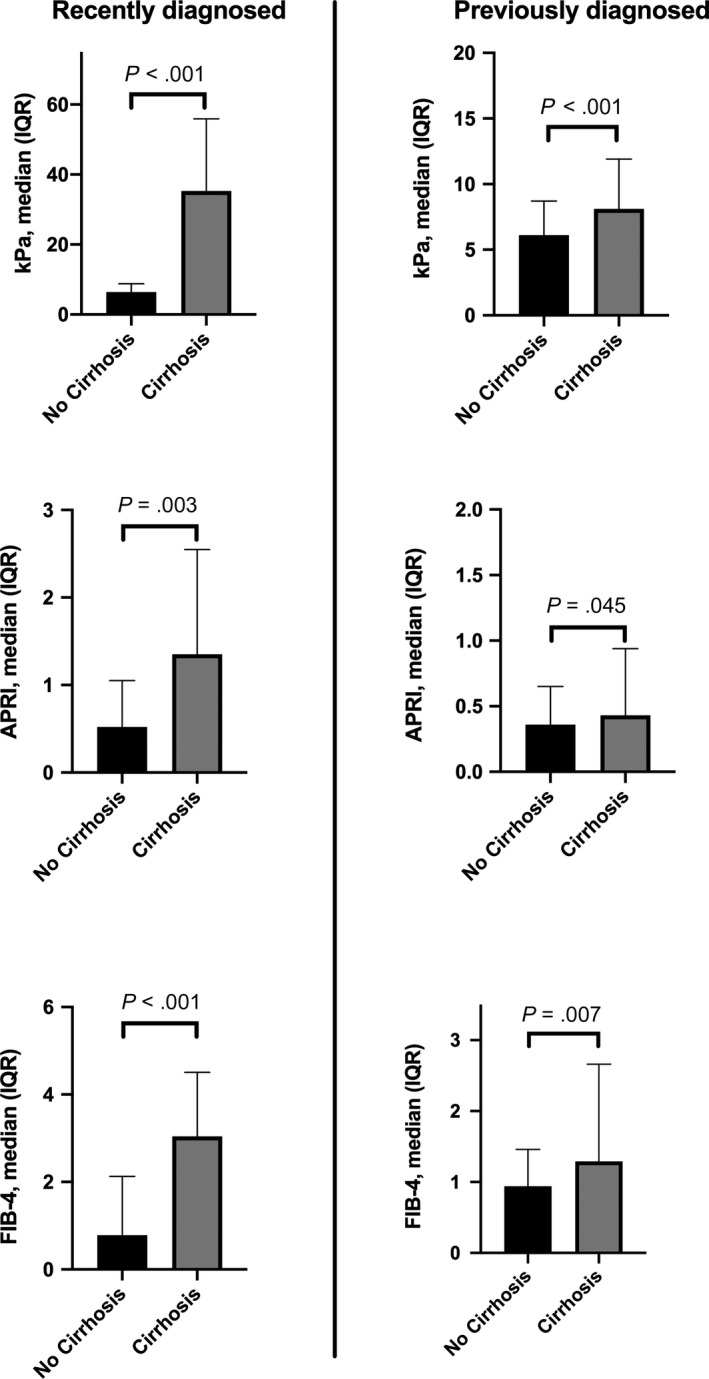
Median TE, APRI and FIB‐4 levels in recently diagnosed and previously diagnosed patients. TE, transient elastography

The Area Under the Curve (AUC) for diagnosing cirrhosis by LSM using TE was 0.76 (0.68‐0.84, *P* < .001; Optimal Cut‐off ≥9.9 kPa: Sensitivity 61%, Specificity 85%, PPV: 61%, NPV: 85%) in all patients irrespective of treatment status (Table 2). In recently diagnosed patients the AUC for correctly detecting cirrhosis using LSM was 0.96 (95% CI: 0.90‐1.0, *P* < .001; Cut‐Off ≥9.9 kPa, PPV: 74%, NPV 100%, Sensitivity 100%, Specificity 83%). Diagnostic accuracy of LSM significantly declined the longer LBX and initial LSM were apart (TE 1‐5 years from LBX: AUC 0.78 [95% CI: 0.59‐0.97], *P* = .023, PPV: 56%, NPV: 85%; Sensitivity 63%, Specificity 81%; TE 5‐10 years from LBX: AUC 0.63 [95% CI: 0.43‐0.83], *P* = .264, PPV: 50%, NPV: 80%, Sensitivity 25%, Specificity 92%; Table 2) and the AUC for detecting cirrhosis in any patient that was classified as “previously diagnosed” was 0.70 (95% CI: 0.60‐0.80, *P* < .001; PPV: 53%, NPV: 82%, Sensitivity: 46%, Specificity: 86%). When all patients with transaminase levels (ALT or AST) ≥2x ULN were excluded from analysis diagnostic accuracy stayed stable or declined marginally (Table S4). LSM was significantly higher in cirrhotic vs non‐cirrhotic patients [All: 11.3 vs 6.1 kPa, *P* < .001]. Median LSM values were 35.3 kPa (22.5‐55.9) in recently diagnosed cirrhotics and this was significantly higher than in recently diagnosed non‐cirrhotics (6.4 kPa [5.3‐8.8], *P* < .001, Figure 2). The optimal Youden Index‐derived LSM cut‐off for detection of cirrhosis in recently diagnosed patients was at ≥9.9 kPa (Sensitivity 100%, Specificity 83.3%, PPV: 73.7% NPV: 100%).

**Table 2:** Diagnostic accuracy for histological cirrhosis for (A) LSM by TE and for (B) serum fibrosis scores (APRI, FIB‐4)

	N	Cut‐off	PPV	NPV	Sensitivity	Specificity
LSM, all patients	188	≥9.9 kPa	61%	85%	61%	85%
APRI, all patients	182	>1.5	57%	76%	24%	93%
FIB‐4, all patients	182	>3.25	65%	77%	26%	95%
A						
Recently (<1 y) diagnosed	44	≥9.9 kPa	73.7%	100%	100%	83.3%
Previously diagnosed	144	≥9.9 kPa	53%	82%	46%	86%
1‐5 y	29	≥9.9 kPa	56%	85%	63%	81%
5‐10 y	34	≥9.9 kPa	50%	80%	25%	92%
B						
Recently (<1 y) Diagnosed	42					
APRI		>1.5	55%	74%	43%	82%
FIB‐4		>3.25	75%	77%	43%	93%
Previously diagnosed	140					
APRI		>1.5	60%	77%	17%	96%
FIB‐4		>3.25	58%	77%	19%	95%
1‐5 y	29					
APRI		>1.5	33%	73%	13%	91%
FIB‐4		>3.25	33%	73%	13%	91%
5‐10 y	32					
APRI		>1.5	33%	79%	14%	92%
FIB‐4		>3.25	100%	81%	14%	100%

Abbreviation: LSM, liver stiffness measurement.

### 3.2 Diagnostic accuracy of APRI and FIB‐4 to diagnose cirrhosis

AUC for diagnosing cirrhosis was 0.67 (Cut‐Off: >1.5, PPV: 57%, NPV: 76%, Sensitivity: 24%, Specificity 93%) and 0.71 (Cut‐Off: >3.25, PPV: 65%, NPV: 77%, Sensitivity: 26%, Specificity: 95%) for APRI/FIB‐4 in all patients (Table 2). In recently diagnosed patients the AUC for correctly detecting cirrhosis using non‐invasive fibrosis scores was 0.79 (0.65‐0.93, *P* = .003; PPV: 55%, NPV: 74%, Sensitivity: 43%, Specificity: 82%) for APRI and 0.84 (0.83‐0.96, *P* < .001; PPV: 65%, NPV: 77%, Sensitivity: 26%, Specificity: 95%) for FIB‐4. Similar to LSM, AUC significantly declined the longer LBX and APRI/FIB‐4 were apart (APRI: 1‐5 years: AUC 0.68 [0.46‐0.89], p0.143 ‐ 5‐10 years: AUC 0.55 [0.31‐0.79] *P* = .698/FIB‐4: 1‐5 years: AUC 0.67 [0.43‐0.91], *P* = .172 ‐ 5‐10 years: AUC 0.62 [0.42‐0.83], *P* = .327, Table 2 and Table S4) and the AUC for detecting cirrhosis in any previously diagnosed patients was 0.61 (0.51‐0.72, *P* = .045; PPV:60%, NPV: 77%, Sensitivity: 17%, Specificity: 96%) for APRI and 0.65 (0.55‐0.75, *P* = .007; PPV: 58%, NPV: 77%, Sensitivity: 19%, Specificity: 95%) for FIB‐4, hence clearly lower than in recently diagnosed patients. When patients with transaminase levels ≥2xULN were excluded AUC by trend increased or stayed stable (Table S4). APRI and FIB‐4 scores were significantly higher in both recently diagnosed and previously diagnosed patients with cirrhosis (Table 4). Conventional cut‐offs to determine advanced fibrosis/cirrhosis for APRI (>1.5) and FIB‐4 (>3.25) correctly identified only 24% and 26% within all patients, however 93% (APRI) and 95% (FIB‐4) of patients without cirrhosis were correctly ruled out. In recently diagnosed patients 82% and 93% of patients with cirrhosis were correctly ruled out using the conventional APRI (Sens. 43%, Spec. 82%, PPV 55%, NPV 74%) and FIB‐4 (Sens. 43%, Spec. 93%, PPV 75%, NPV 77%)cut‐offs.

### 3.3 Factors influencing LSM in WD patients

Binary logistic regression analysis was used to highlight factors that directly influence LSM in patients with WD (Table 3). On univariable analysis male gender (OR 0.47, *P* = .023 for being female), cirrhosis at liver biopsy (OR 9.07, *P* < .001), decreasing platelet count (OR 0.98, *P* < .001), increasing APRI (OR 3.74, *P* < .001) and FIB‐4 (OR 2.28, *P* < .001) as well as treatment status at time of LSM (OR 0.38, *P* = .007 for being under treatment at LSM) were associated with LSM values ≥9.9 kPa. In multivariable analyses (Model 1: APRI, Model 2: FIB‐4) male gender, cirrhosis at LBX and APRI respectively FIB‐4 were independently associated with LSM values ≥9.9 kPa (Table 3).

**Table 3:** Determinants of LSM values ≥ 9.9 kPa in patients with WD (univariable and multivariable binary regression analyses)

	Univariable			Multivariable‐Model APRI			Multivariable‐Model FIB‐4		
	OR	95%CI	*P*‐value	OR	95%CI	*P*‐value	OR	95%CI	*P*‐value
Age	1.02	0.99‐1.04	.097	1.03	0.99‐1.06	.066	—	—	—
Gender (being female)	0.47	0.24‐0.90	.023	0.26	0.11‐0.65	.004	0.30	0.12‐0.76	.011
Cirrhosis on LBX	9.07	4.35‐18.92	<.001	9.17	3.62‐23.26	<.001	9.70	3.87‐24.28	<.001
AST	1.00	0.99‐1.01	.106	—	—	—	—	—	—
ALT	1.00	0.99‐1.01	.209	—	—	—	—	—	—
PLT	0.98	0.97‐0.99	<.001	—	—	—	—	—	—
APRI^a^	3.74	2.15‐6.53	<.001	3.25	1.71‐6.19	<.001	—	—	—
FIB‐4^a^	2.28	1.66‐3.12	<.001	—	—	—	1.83	1.30‐2.59	.001
Treatment >1 y at time of first LSM (OR for being under treatment at first TE)	0.38	0.18‐0.77	.007	0.75	0.26‐2.13	.584	0.57	0.21‐1.49	.248

Abbreviations: LSM, liver stiffness measurement; TE, transient elastography; WD, Wilson disease.

a Available in 182 patients.

### 3.4 LSMs and non‐invasive fibrosis scores during long‐term follow‐up

**Figure 3:**
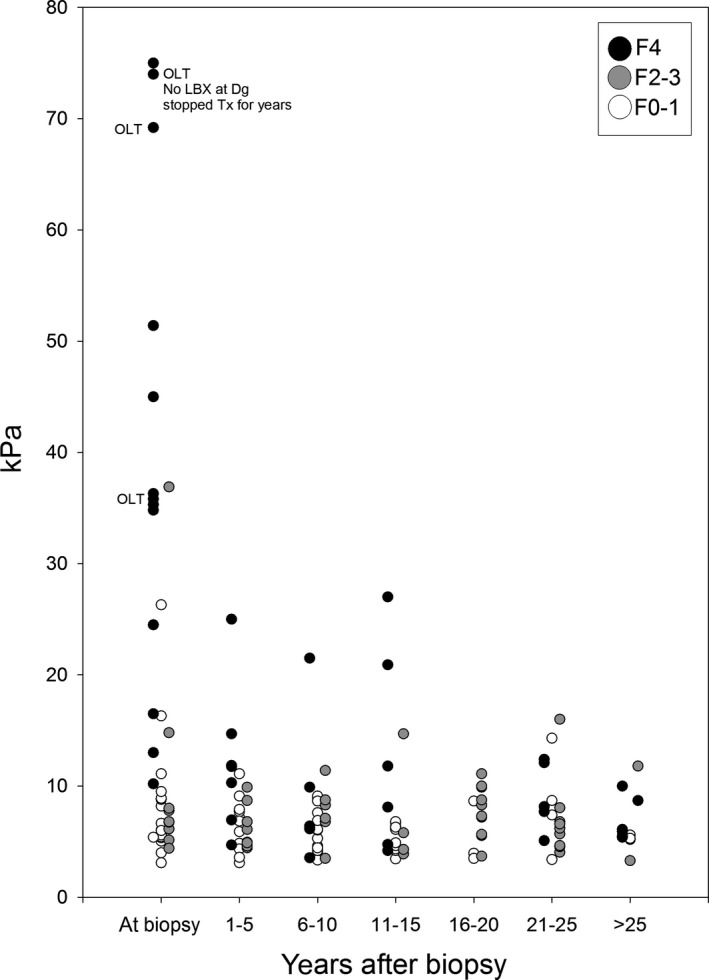
Time (years) after LBX when LSM was performed. OLT (orthotopic liver transplantation) refers to patients that have been ‘high urgently’ transplanted shortly after LBX and LSM have been performed. LSM, liver stiffness measurement

**Figure 4:**
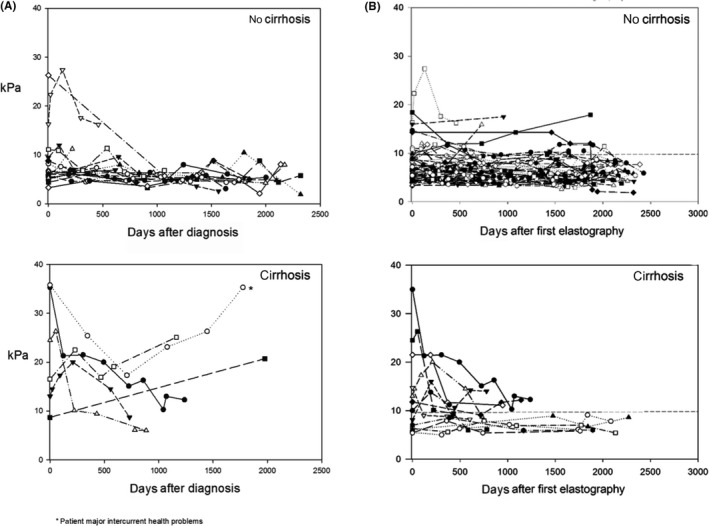
(A) LSM over time in recently diagnosed patients, stratified according to presence/absence of cirrhosis (B) LSM over time in previously diagnosed patients, stratified according to presence/absence of cirrhosis. LSM, liver stiffness measurement

At least one LSM during follow‐up was available in 128 patients (68.1%; 1xLSM: 44 [23.4%], 2xLSM: 21 [11.2%], 3xLSM: 13 [6.9%], 4xLSM: 15 [8.0%], 5xLSM: 14 [7.5%], 6xLSM: 10 [5.3%], 7xLSM: 6 [3.2%], 8xLSM: 5 [2.7%]; Figure 3). Forty‐eight (37.5%) patients showed improved (at least −10% and/or −1 kPa), 38 (29.7%) stable (±10%) and 42 (32.8%) increased (at least +10% and/or +1 kPa) last LSM compared to BL‐LSM levels (Figures 1 and 4). In total, 8/100 (8%) patients with LSM <9.9 kPa at first measurement increased over the 9.9 kPa threshold and 13/28 (46.4%) with LSM ≥9.9 at first measurement decreased below the 9.9 kPa threshold. During a median follow‐up of 46 (24‐66) months, 5.9% (5/85) of non‐cirrhotic WD patients showed progression to cirrhotic FU‐LSM values, while 30.8% (4/13) of cirrhotic WD patients had FU‐LSM suggestive of cirrhosis regression.

In recently diagnosed non‐cirrhotic patients median first LSM was 6.4 kPa and median last LSM was 5.7 kPa (*P* = .076, Table 4). In recently diagnosed cirrhotic patients median first LSM was 35.3 kPa and median last LSM 18.7 kPa (*P* = .116, Table 4). Overall, in recently diagnosed patients, 1 of 18 (5.6%) with LSM <9.9 kPa at first measurement increased above 9.9 kPa (reason: concomitant non‐alcoholic steatohepatitis as a result of adipositas per magna) and 4 of 9 (44.4%) with LSM ≥9.9 kPa decreased below 9.9 kPa (Figure 4A) at last LSM.

**Table 4:** Dynamics of transient elastography values and non‐invasive fibrosis scores during follow‐up in recently diagnosed patients

	First LSM	Last LSM	*P*‐value	Time period from fist to last LSM (mo)
Non‐cirrhotic patients				
kPa, median (IQR)	6.4 (5.3‐8.8) n = 30	5.7 (4.9‐7.6) n = 21	.076	53.5 mo (22.3‐67.9)
APRI, median (IQR)	0.52 (0.28‐1.05) n = 28	0.53 (0.27‐0.80) n = 19	.159	
FIB‐4, median (IQR)	0.78 (0.44‐2.13) n = 28	0.60 (0.45‐1.18) n = 19	.573	
Cirrhotic patients				
kPa, median (IQR)	35.3 (22.5‐55.9) n = 14	18.7 (8.1‐29.1) n = 6	.116	31.8 mo (18.3‐45.0)
APRI, median (IQR)	1.35 (0.94‐2.55) n = 14)	0.78 (0.33‐1.42) n = 6	.075	
FIB‐4, median (IQR)	3.04 (1.72‐4.51) n = 14	2.14 (1.05‐5.13) n = 6	.345	

Abbreviation: LSM, liver stiffness measurement.

In previously diagnosed patients last LSM was not different from baseline (cirrhosis: 8.1 vs 7.4 kPa; *P* = .897; non‐cirrhotic: 6.1 vs 6.2 kPa; *P* = .899, Figure 4, Table S1). Overall, LSM increased above 9.9 kPa in 7/82 (8.5%) non‐cirrhotic patients at last testing. Furthermore 9/19 (47.4%) patients with initial values ≥9.9 kPa fell below the 9.9 kPa threshold at last‐LSM (Figure 4B).

Overall, in recently diagnosed patients baseline ALT (n = 27) was 40 U/L (26‐109) and last available ALT was 34 U/L (22‐52; *P* = .990), in those with cirrhosis absolute ALT values tended to decline (baseline: 54 U/L [25‐101] vs 25 U/L [21‐38], *P* = .116). In the four patients with a decrease of the last LSM values below the 9.9 kPa cut‐off, median ALT levels were 74 (44‐211) at BL vs 26 (21‐131) at last‐FU (*P* = .068). In the patient (BL‐ALT: 44 IU/mL) with an increase of LSM over the threshold, last‐ALT was 110.

## 4 DISCUSSION

There is a need for non‐invasive parameters to monitor the outcome of chronic liver diseases overall, and in patients with WD in particular. Because of the relative rarity of WD there are no prospective longitudinal studies available, however there is an urgent need for strategies to monitor treatment and define endpoints for Phase 3 trials. With all the limitations of our study three major conclusions can be drawn from the data obtained in this large cohort of WD patients with liver biopsy: (a) in recently diagnosed patients LSM using TE is a useful tool identify patients with cirrhosis and (b) in previously diagnosed patients fibrosis remained stable or may have even regressed (c) in previously diagnosed and recently diagnosed patients around 8.5% and 5.6% worsened under therapy.

Calculation of a cut‐off value for cirrhosis in WD is limited because of the small sample size. However, it should be stressed that 44 recently diagnosed adult patients with a liver biopsy at diagnosis in a rare disease is quite large. The calculated LSM cut‐off for diagnosis of cirrhosis in recently diagnosed WD patients of ≥9.9 kPa showed excellent sensitivity (100%) and specificity (83.3%) respectively. One could argue that this cut‐off is lower than cut‐offs reported in other aetiologies.^9^, ^13^, ^20^ The cut‐off for cirrhosis in chronic hepatitis C is not well–defined; values in the literature range between 10 and 17.6 kPa.^13^ Overall, TE values <10 kPa usually rule out compensated advanced chronic liver disease and values between 10 and 15 kPa indicate compensated advanced chronic liver disease whereas values >15 kPa are highly indicative of compensated advanced chronic liver disease.^21^ This is also in accordance with our data, where we found an NPV of 100% for the cut‐off of 9.9 kPa in recently diagnosed WD patients. Regarding non‐invasive laboratory‐based scores, APRI and FIB‐4 were both useful to rule out advanced liver disease rather than diagnosing cirrhosis. They could therefore in addition to LSM help identify/ruling out cirrhotic WD patients.

A large proportion of patients had repeated TE measurements during follow‐up and we could show that in non‐cirrhotic patients only a minority progressed to cirrhosis specific FU‐LSM values (5.9%) and 30.8% of cirrhotic patients even regressed to FU‐LSM values <9.9 kPa, suggestive of cirrhosis regression.

Our data indicate little disease progression and regression of LSM, APRI and FIB‐4 in most of the patients under therapy and suggest LSM and non‐invasive fibrosis markers as valuable tools for disease monitoring in WD. Even though a clear trend towards regression is shown by absolute numbers (Table 4, Tables S1 and S2, Figure 4) our results in this regard did not attain statistical significance and this can probably be explained by an underpowered study, which however comprises a bias that because of the rarity of WD can hardly be overcome. Nevertheless the results are in line with clinical studies showing that once therapy is initiated most patients have a stable disease, or even improve.^22^ Furthermore the lower frequency of oesophageal varices and the low TE values in cirrhotic patients on treatment suggest that most of these patients had stable liver disease and in some fibrosis may have decreased. Accordingly, long‐term follow‐up data found no survival difference in adequately treated WD patients compared to the general population^23^ and death‐rates and liver‐related mortality were low.^24^, ^25^, ^26^, ^27^, ^28^ Similarly, studies evaluating TE pre‐ and post‐HCV therapy, also showed a marked decrease of TE values in patients who achieved sustained virologic response.^29^, ^30^, ^31^, ^32^, ^33^ It is possible that treatment reduced inflammatory components like in chronic hepatitis C, but not the structural alterations of fibrosis or cirrhosis. TE results may be influenced by ALT flares.^34^ In our cohort ALT values declined in all four recently diagnosed patients where LSM declined below the 9.9 kPa threshold at Last‐FU, further suggesting a possible disease regression in those patients.

The strengths of our study definitely are the large number of patients in a rare disease, availability of histological data on every patient and data on treatment status. This is the first study presenting cut‐offs for ruling in/out cirrhosis in the largest cohort of recently diagnosed and on treatment WD patients so far and we presented cut‐off for TE, APRI and FIB‐4 that might help ruling in/out cirrhosis in WD in daily clinical practice. Furthermore this is the first study reporting longitudinal LSM data in a sufficient number of WD patients. The largest limitation is the retrospective analysis of biopsy data, the lack of a central hepatopathologist and the small sample size of longitudinal data. Although LSM was done prospectively as part of the clinical routine, it is a retrospective analysis and not a prospectively planned study. There may have been interobserver differences between the three different centres, which are very unlikely, since TE examinations were performed by experienced testers using the same equipment. The widely established quality criteria for LSM measurements^21^ were exactly followed. The most severe cases with the highest LSM underwent transplantation shortly after diagnosis and thus, the results regarding follow‐up in de‐novo patients warrant cautious interpretation unless more data on follow‐up LSM are available.

In conclusion in recently diagnosed patients with WD, cirrhosis can be suggested if LSM values are ≥9.9 kPa. Furthermore APRI and FIB‐4 seem to be feasible tools to rule out cirrhosis. Once treatment is initiated LSM values and non‐invasive fibrosis scores appear to decline or remain stable. Thus, repeated measurements of LSM might be useful to evaluate long‐term treatment effects on hepatic WD. The availability of non‐invasive markers is not only useful to long‐term monitoring of patients, but will become essential for the design of treatment trials.

## CONFLICT OF INTEREST

The authors do not have any disclosures to report.

## AUTHOR CONTRIBUTIONS

All authors contributed either to the research design (RP, AF, TR, KHW and PF), and/or the acquisition (clinical data: RP, AF AFS, JP, RS, PF, TR and KHW, histological data: RP, PF, KHW, FW, TL and KL), analysis (RP, TR and PF) or interpretation (all authors) of data. RP, TR and PF drafted the manuscript, which was then critically revised by all other authors. All authors approved the final version of this manuscript.
