# Supplementary material for: Non‐invasive diagnosis of cirrhosis and long‐term disease monitoring by transient elastography in patients with Wilson disease
Source: Liver Int. 2020 Jan 22;40(4):894–904. doi: 10.1111/liv.14368 (PMC7187206; doi:10.1111/liv.14368)
Supplement: Supplementary file 1 [file LIV-40-894-s001.docx]

**Supplemental Table T1: Median TE values during follow-up grouped according to treatment-status and presence/absence of cirrhosis LBX.**

|  | **BL-TE** | **Last-TE** | **Last vs. BL** | **FU-1** | **FU-1 vs. BL** | **FU-2** | **FU-2 vs BL** | **FU-3** | **FU-3 vs. BL** | **FU-4** | **FU-4 vs BL** | **FU-5** | **FU-5 vs. BL** | **FU-6** | **FU-6 vs. Bl** | **FU-7** | **FU-7 vs. BL** | **FU-8** | **FU-8 vs. BL** |
| --- | --- | --- | --- | --- | --- | --- | --- | --- | --- | --- | --- | --- | --- | --- | --- | --- | --- | --- | --- |
| ***Newly diagnosed cirrhotic patients*** | | | | | | | | | | | | | | | | | | | |
| **kPa (IQR)**  **N(%)** | 35.3 (22.5-55.9)  14 (100%) | 18.7 (8.1-29.1)  6 (43%) | 0.116 | 23.4 (18.0-26.5)  6 (43%) | 0.345 | 17.3 (13.7-23.3)  5 (36%) | 0.225 | 20 (12.1-22.3)  4 (29%) | 0.144 | 14.8 (8.1-23.5)  4 (29%) | 0.144 | 12.5 (6.8-30.6)  4 (29%) | 0.068 | 10.3  1 (7%) | - | 13.0  1 (7%) | - | 12.3  1 (7%) | - |
| ***Newly diagnosed non-cirrhotic patients*** | | | | | | | | | | | | | | | | | | | |
| **kPa (IQR)**  **N(%)** | 6.4 (5.3-8.8)  30 (100%) | 5.7 (4.9-7.6)  21 (70%) | 0.076 | 6.9 (5.4-9.6)  21 (70%) | 0.297 | 5.6 (4.7-6.7)  16 (53%) | 0.103 | 5.8 (5.3-6.6)  14 (47%) | 0.489 | 5.0 (4.6-9.2)  13 (43%) | 0.294 | 6.1 (4.6-8.5)  9 (30%) | 0.484 | 4.9 (4.3-5.9)  8 (27%) | 0.018 | 5.1 (4.0-6.9)  5 (17%) | 0.225 | 5.0  3 (10%) | 0.109 |
| ***Pre-treated cirrhotic patients*** | | | | | | | | | | | | | | | | | | | |
| **kPa (IQR)**  **N(%)** | 8.1 (6.0-11.9)  37 (100%) | 7.4 (5.2-11.8)  22 (60%) | 0.897 | 8.2 (4.7-11.9)  22 (60%) | 0.852 | 6.7 (5.7-10.0)  13 (35%) | 0.784 | 8.2 (6.5-10.9)  9 (24%) | 0.859 | 7.0  3 (8%) | 0.109 | 9.1  3 (8%) | 0.285 | 7.8  1 (3%) | - | -  0 (0%) | - | -  0 (0%) | - |
| ***Pre-treated non-cirrhotic patients*** | | | | | | | | | | | | | | | | | | | |
| **kPa (IQR)**  **N(%)** | 6.1 (4.6-8.7)  107 (100%) | 6.2 (4.5-8.0)  79 (74%) | 0.899 | 6.1 (4.8-9.2)  79 (74%) | 0.335 | 5.8 (4.5-7.6)  50 (47%) | 0.269 | 5.5 (4.4-8.3)  36 (34%) | 0.514 | 6.0 (5.2-7.9)  30 (28%) | 0.067 | 7.6 (5.1-8.4)  19 (18%) | 0.235 | 5.7 (3.9-7.1)  11 (10%) | 0.657 | 7.2 (5.2-8.2)  5 (5%) | 0.225 | 8.5  1(0.9%) | - |

- P-value not possible due to small sample-size

**Supplemental Table T2: Median APRI and FIB-4 values during follow-up in newly diagnosed patients after treatment was started.**

|  | **BL** | **Last-FU** | **Last vs. BL** | **FU-1** | **FU-1 vs. BL** | **FU-2** | **FU-2 vs BL** | **FU-3** | **FU-3 vs. BL** | **FU-4** | **FU-4 vs BL** | **FU-5** | **FU-5 vs. BL** | **FU-6** | **FU-6 vs. Bl** | **FU-7** | **FU-7 vs. BL** | **FU-8** | **FU-8 vs. BL** |
| --- | --- | --- | --- | --- | --- | --- | --- | --- | --- | --- | --- | --- | --- | --- | --- | --- | --- | --- | --- |
| ***Newly diagnosed cirrhotic patients*** | | | | | | | | | | | | | | | | | | | |
| **APRI**  **FIB-4**  **N(%)** | 1.35 (0.94-2.55)  3.04 (1.72-4.51)  14 (100%) | 0.79 (0.33-1.42)  2.14 (1.05-5.13)  6 (43%) | 0.075  0.345 | 1.17 (0.74-1.42)  2.66 (1.86-4.15)  6 (43%) | 0.075  0.116 | 0.87 (0.43-1.26)  2.03 (1.11-4.22)  4 (29%) | 0.273  0.465 | 0.48 (0.45-1.46)  1.45 (1.03-5.14)  4 (29%) | 0.465  0.715 | 0.57 (0.38-1.56)  1.73 (0.94-5.78)  4 (29%) | 0.465  0.715 | 0.42 (0.29-0.96)  1.22 (0.81-4.84)  4 (29%) | 0.144  0.715 | 0.40  1.56  1 (7%) | - | 0.36  1.39  1 (7%) | - | 0.36  1.66  1 (7%) | - |
| ***Newly diagnosed non-cirrhotic patients*** | | | | | | | | | | | | | | | | | | | |
| **APRI**  **FIB-4**  **N(%)** | 0.52 (0.28-1.05)  0.78 (0.44-2.13)  28 (100%) | 0.53 (0.27-0.80)  0.60 (0.45-1.18)  19 (68%) | 0.159  0.573 | 0.32 (0.21-0.75)  0.50 (0.38-0.73)  21* (75%) | 0.064  0.049 | 0.41 (0.30-0.67)  0.61 (0.46-0.89)  16 (57%) | 0.281  0.865 | 0.31 (0.23-0.48)  0.59 (0.46-0.87)  14 (50%) | 0.087  0.311 | 0.29 (0.23-0.40)  0.61 (0.44-0.99)  13 (46%) | 0.019  0.209 | 0.33 (0.22-0.39)  0.50 (0.43-0.87)  9 (32%) | 0.161  0.575 | 0.34 (0.23-0.51)  0.63 (0.46-0.94)  8 (29%) | 0.735  0.612 | 0.35 (0.24-0.59)  0.53 (0.41-1.78)  5 (18%) | 0.465  0.273 | 0.33  0.64  3 (10%) | 0.180  0.180 |

- P-value not possible due to small sample-size

* Two patients had missing baseline APRI/FIB-4 scores

**Supplemental Table T3: Clinical characteristics of newly diagnosed an pre-treated patients, stratified according to their diagnosis at liver biopsy (cirrhosis vs. no-cirrhosis).**

|  | **Newly diagnosed (n=44)** | | | **Under-treatment (n=144)** | | |
| --- | --- | --- | --- | --- | --- | --- |
|  | **No cirrhosis (n=30)** | **Cirrhosis (n=14)** | **p-value** | **No cirrhosis (n=137)** | **Cirrhosis (n=37)** | **p-value** |
| **Age, mean+SD** | 28±14 | 34±9 | 0.115 | 34±13 | 41±14 | 0.008 |
| **Sex, n(%)**  **Male**  **Female** | 16(66.7%)  14(70.0%) | 8 (33.3%)  6 (30.0%) | 0.813 | 49(80.3%)  58(69.9%) | 12(19.7%)  25(30.1%) | 0.156 |
| **kPa, median (IQR)** | 6.4 (5.3-8.8) | 35.3 (22.5-55.9) | <0.001 | 6.1 (4.6-8.7) | 8.1 (6.0-11.9) | <0.001 |
| **kPa-grouped, n(%)**  **<10 kPa**  **≥10 kPa** | 25 (100%)  5 (26.3%) | 0 (0%)  14(73.7%) | <0.001 | 92(82.1%)  15(46.9%) | 20(17.9%)  17(53.1%) | <0.001 |
| **APRI, median (IQR)*** | 0.52 (0.28-1.05) | 1.35 (0.94-2.55) | 0.003 | 0.36 (0.22-0.65) | 0.43 (0.28-0.94) | 0.045 |
| **APRI- grouped, n(%)**  **<1.5**  **>1.5** | 23(74.2%)  5 (45.5%) | 8 (25.8%)  6 (54.5%) | 0.082 | 100(76.9%)  4 (40%) | 30(23.1%)  6 (60%) | 0.010 |
| **FIB-4, median (IQR)*** | 0.78 (0.44-2.13) | 3.04 (1.72-4.51) | <0.001 | 0.94 (0.56-1.46) | 1.29 (0.85-2.66) | 0.007 |
| **FIB-4 – grouped, n(%)**  **<3.25**  **>3.25** | 26(76.5%)  2 (25.0%) | 8 (23.5%)  6 (57.1%) | 0.005 | 99(77.3%)  5 (41.7%) | 29(22.7%)  7 (58.3%) | 0.007 |

- **Available in 182 patients.**

**Supplemental Table T4: Diagnostic accuracy for histological cirrhosis for (A) LSM by TE and for (B) serum fibrosis scores (APRI, FIB-4).**

|  | **N** | **All patients included** | | | **TAs ≥ 2x ULN excluded** | | |
| --- | --- | --- | --- | --- | --- | --- | --- |
|  |  | **AUC** | **95%CI** | **p-value** | **AUC** | **95%CI** | **p-value** |
| **LSM, all patients** | 188 | 0.76 | 0.68-0.84 | <0.001 | 0.72  (n=153) | 0.63-0.81 | <0.001 |
| **APRI, all patients** | **182** | 0.67 | 0.58-0.76 | <0.001 | 0.69  (n=151) | 0.60-0.79 | <0.001 |
| **FIB-4, all patients** | **182** | 0.71 | 0.63-0.80 | <0.001 | 0.70  (n=151) | 0.61-0.79 | <0.001 |
| **A** |  | **AUC** | **95% CI** | **p-value** | **AUC** | **95%CI** | **p-value** |
| **Recently (<1y) diagnosed** | **44** | 0.96 | 0.90-1.0 | <0.001 | 0.92*^1^ | 0.81-1.0 | 0.001 |
| **all treated** | 144 | 0.70 | 0.60-0.80 | <0.001 | 0.68^##1^ | 0.58-0.79 | 0.002 |
| **1-5y** | **29** | 0.78 | 0.59-0.97 | 0.023 | 0.75**^1^ | 0.54-0.96 | 0.057 |
| **5-10y** | 34 | 0.63 | 0.43-0.83 | 0.264 | 0.64^#1^ | 0.42-0.86 | 0.268 |
| **B** |  | **AUC** | **95% CI** | **p-value** | **AUC** | **95%CI** | **p-value** |
| **Recently (<1y) Diagnosed**  **APRI**  **FIB-4** | 42 | 0.79  0.84 | 0.65-0.93  0.73-0.96 | 0.003  <0.001 | 0.86*^2^  0.87 | 0.70-1.0  0.73-1.0 | 0.004  0.003 |
| **Under treatment**  **APRI**  **FIB-4** | **140** | 0.61  0.65 | 0.51-0.72  0.55-0.75 | 0.045  0.007 | 0.66^##2^  0.66 | 0.55-0.77  0.56-0.77 | 0.006  0.006 |
| **1-5y**  **APRI**  **FIB-4** | **29** | 0.68  0.67 | 0.46-0.89  0.43-0.91 | 0.143  0.172 | 0.73^**2^  0.66 | 0.50-0.96  0.40-0.92 | 0.079  0.226 |
| **5-10y**  **APRI**  **FIB-4** | **32** | 0.55  0.62 | 0.31-0.79  0.42-0.83 | 0.698  0.327 | 0.64^#2^  0.69 | 0.39-0.88  0.48-0.89 | 0.293  0.150 |

*^1^n=30 , ^*2^n=28

**^1^n=25, ^**2^n=25

^#1^n=27, ^#2^n=27

^##1^n=123, ^##2^n=123
